# Dorsal Arachnoid Web as an Uncommon Cause of Thoracic Myelopathy: A Case Report

**DOI:** 10.51894/001c.165977

**Published:** 2026-07-31

**Authors:** Paige Norwood, Drew Parkhurst

**Affiliations:** 1 College of Osteopathic Medicine Michigan State University

## 108

### Introduction

Thoracic myelopathy is an uncommon but potentially disabling spinal cord disorder with a myriad of etiologies. Degenerative changes leading to central and neuroforaminal stenosis are frequently identified on imaging of older patients; however, there are rare intradural pathologies that may lead to similar clinical presentations. We describe a case of a unique etiology of thoracic myelopathy due to a dorsal arachnoid web or cyst leading to focal cord compression and myelomalacia.

### Case Description

A 68-year-old male with a history of arthritis, hypertension, and intellectual disability presented with chronic midline thoracic pain progressively worsening since 1981 at which time he had an occupation requiring heavy lifting. His pain was located between the scapulae with radiation to bilateral lower extremities. He had worsening balance/coordination that led to the use of cane for ambulation. He denied symptoms of bowel or bladder dysfunction, saddle anesthesia, or lower extremity weakness. He had tried conservative measures including physical therapy and activity modification with minimal pain relief. Physical exam revealed 5/5 strength in lower extremities, down-going Babinski reflexes, 3+ patellar reflexes, and 2+ achilles reflexes symmetric bilaterally. FADIR, hip log roll, and straight leg raise tests were negative bilaterally, but did cause reproduction of thoracic pain. He had tenderness to palpation of thoracic spinous processes of T6-9. Initial MRI without contrast of the thoracic spine demonstrated cord signal change at T7–8 suggestive of myelomalacia without a clear compressive lesion. Follow-up MRI with contrast re-demonstrated focal right hemicord myelomalacia at T7–8 and posterior cord scalloping with preserved ventral cerebrospinal fluid space, suspicious for a dorsal arachnoid web versus cyst. He had previously been evaluated by neurosurgery in Michigan that recommended non-operative management; however, the patient and family requested a referral to Cleveland Clinic that later performed a decompression with resolution of symptoms.

### Discussion

This case highlights how recognizing these uncommon etiologies of thoracic myelopathy including dorsal arachnoid webs and cysts is critical and should be considered when imaging reveals changes in cord signal without obvious degenerative compression or mass. Surgical decompression may halt further neurologic decline and can lead to significant symptomatic improvement.

